# Refractory exudative pleural effusion in patients with chronic kidney disease not receiving dialysis: A case report

**DOI:** 10.1002/ccr3.2069

**Published:** 2019-02-19

**Authors:** Hye Mi Seo, Miyeon Kim, Hyunwoo Kim

**Affiliations:** ^1^ Division of Nephrology, Department of Internal Medicine, School of Medicine, Jeju National University Hospital Jeju National University Jeju City Korea

**Keywords:** chronic kidney disease, corticosteroid, exudative pleural effusion

## Abstract

Although exudative pleural effusion can be caused by infections, malignancies, and connective tissue diseases, we need to consider uremic pleural effusion and pleuritis in differential diagnosis of exudative lymphocyte predominant pleural effusion in patients with chronic kidney disease not receiving dialysis.

## 1 INTRODUCTION

Pleural effusion is a common complication in patients with chronic kidney disease (CKD), particularly in those with end‐stage renal disease (ESRD). Transudative pleural effusion is commonly caused by hypervolemia, whereas exudative pleural effusion can be caused by infections such as tuberculosis, malignancies, and connective tissue diseases.^1^, ^2^, ^3^, ^4^, ^5^ Uremic pleuritis, which is a diagnosis of exclusion and results from inflammation of the visceral and parietal pleura, can also cause exudative pleural effusion.^1^, ^6^, ^7^, ^8^, ^9^ Although the pathogenesis of uremic pleuritis remains uncertain, it has primarily been reported in patients with ESRD receiving dialysis. Intensifying renal replacement regimens is effective in these patients.^1^, ^6^, ^9^, ^10^ Only a few cases of CKD associated pleural effusion have been reported in patients during the predialysis stage, even among those in whom the effusion is expected to resolve after the initiation of dialysis. We describe exudative pleural effusion in a CKD patient not receiving dialysis. The patient was refractory to conventional treatment such as continuous renal replacement therapy but improved completely with corticosteroid therapy.

## 2 CASE EXAMINATION

**Figure 1:**
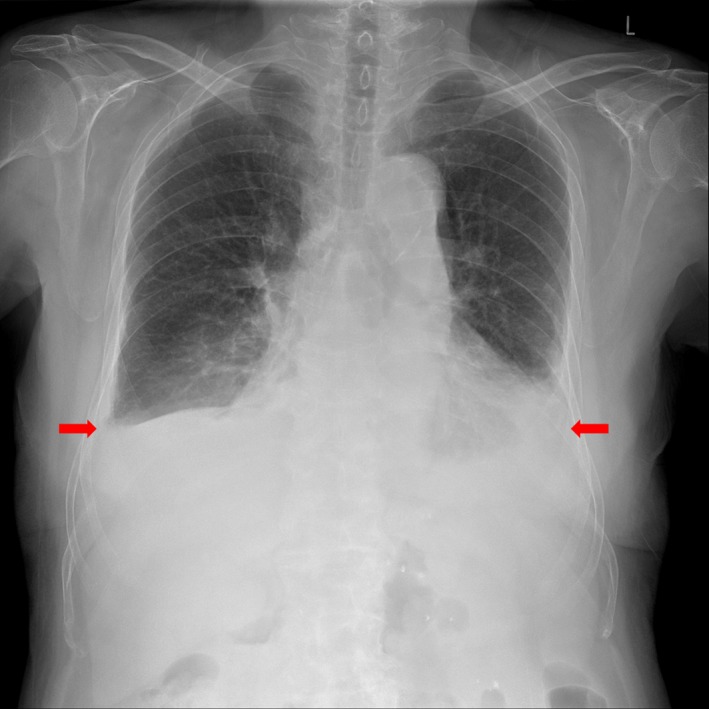
Chest radiograph on admission showing bilateral pleural effusions (arrows)

An 86‐year‐old man, who had suffered from CKD for 5 years probably due to hypertension, presented with shortness of breath for 3 days. He had a 14‐year history of hypertension and a 2‐year history of type 2 diabetes mellitus. He had been taking candesartan 8 mg, teneligliptin 20 mg, and rosuvastatin 5 mg orally once daily. In addition, he had taken some natural products, of which the active components were not identified, for the improvement of renal function from a traditional market for 1 month without consultation with his physician. One month after he had commenced taking the natural products, he developed nausea, loss of appetite, oliguria, and dyspnea, and was admitted to a hospital. His renal function had remained stable for last 1 year. Renal function test performed 4 months earlier revealed a serum creatinine level of 2.70 mg/dL and an estimated glomerular filtration rate (using the Modification of Diet in Renal Disease equation) of 23 mL/min/1.73 m^2^ (stage 4 CKD). On admission, a simple chest X‐ray revealed an increase of bilateral pleural effusions. Laboratory findings showed hypoalbuminemia (2.7 g/dL), worsened renal function (serum creatinine; from 2.70 to 3.51 mg/dL, estimated glomerular filtration rate; from 23 to 17 mL/min/1.73 m^2^), and elevated C‐reactive protein (CRP, 20.8 mg/L). Rheumatoid factor, anticyclic citrullinated peptide antibody, and tumor markers were negative. Sputum was negative for malignant cells and acid‐fast bacilli. On thoracentesis, the pleural effusions were serosanguineous, exudative (pleural fluid to serum protein ratio >0.5 [2.2/4.0 g/dL], pleural fluid to serum lactate dehydrogenase [LDH] ratio >0.6 [164/234 IU/L]), and lymphocyte dominant (87%). Pleural fluid adenosine deaminase level was 20.0 U/L. Based on these results, he was diagnosed with acute kidney injury superimposed on CKD due to ingestion of natural products and exudative pleural effusion unknown etiology. He stopped ingesting natural products and received conservative management including the administration of intermittent albumin and daily furosemide 40 mg intravenously. However, his renal function gradually worsened (serum creatinine; from 3.51 to 4.20 mg/dL), and the increase of effusions accelerated. Finally, furosemide was stopped and he was transferred to our hospital. Upon admission, his blood pressure was 90/51 mm Hg, heart rate was 122 beats/min, respiratory rate was 26 breaths/min, and body temperature was 36.2°C. He further mentioned that he had no history of connective tissue disease such as rheumatoid arthritis and had no fever, night sweat, pleuritic chest pain, purulent sputum production, or weight loss for the last 3 months. Physical examination revealed dull percussion sounds and decreased lung sounds over both lung fields without wheezing or crackles. There was no jugular venous distention on both sides, and cardiac examination was unremarkable. There was no shifting dullness on his abdomen, and his lower extremities showed no pitting edema. There was no other abnormality on his physical examination such as joint swelling and/or tenderness. Chest radiography showed blunting of both costophrenic angles suggesting bilateral pleural effusions (Figure 1).

## 3 DIFFERENTIAL DIAGNOSIS, INVESTIGATIONS, AND TREATMENT

**Figure 2:**
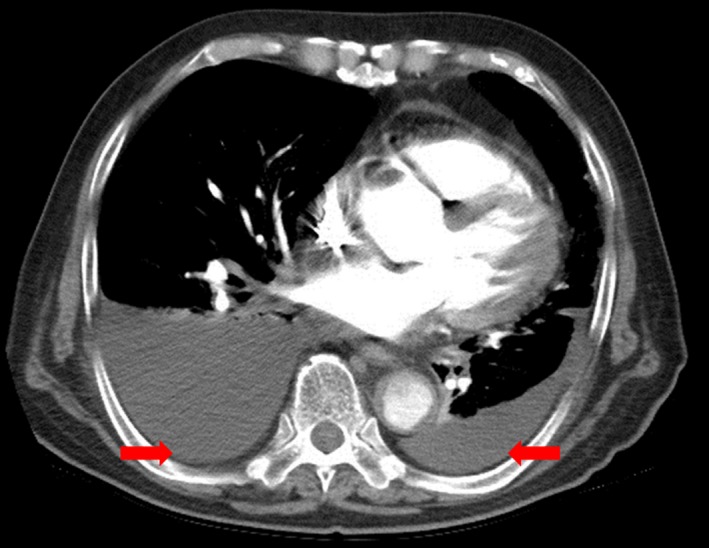
Chest computed tomography on admission showing bilateral pleural effusions (arrows) without any swelling of the lymph nodes

Chest computed tomography (CT) showed a large amount of bilateral effusions without any swelling of the lymph nodes and pulmonary infiltrates (Figure 2). Abdominal CT was not contributory. Echocardiography revealed normal cardiac function (ejection fraction, 60%) without pulmonary hypertension and/or abnormal regional wall motion. There was no significant valvular abnormality and pericardial effusion. Results of laboratory studies showed a serum creatinine level of 4.10 mg/dL, blood urea nitrogen of 45.5 mg/dL, an estimated glomerular filtration rate of 14 mL/min/1.73 m^2^, a leukocyte count of 9600 cells/mm^3^, a hemoglobin level of 9.9 mg/dL, and a platelet count of 119 × 10^3^/mm^3^. The serum CRP level was 30.6 mg/L. The serum total protein and albumin levels were 4.3 and 2.2 g/dL, respectively. However, urinalysis showed no albuminuria. The serum LDH level was 553 IU/L (upper limit of normal value; 460 IU/L). Antinuclear and antineutrophil cytoplasmic antibodies were negative. His serum complement levels (C3 and C4) were also normal. Tests for the human immunodeficiency virus, the hepatitis B and C virus, and SS‐A and SS‐B antibodies were absent. Additionally, thyroid and adrenal panels were normal. Bilateral thoracentesis was performed with drainage of a straw‐colored pleural fluid. Results of pleural fluid analysis carried out simultaneously with blood tests are shown in Table 1. No malignant cells were found in the cytologic examination. Microbiologic smears and cultures of pleural fluid showed no growth. Biochemistry analysis of the pleural fluid was nondiagnostic; therefore, thoracoscopic pleural biopsy was planned. However, the patient refused the procedure owing to his poor general condition. We concerned that he would not be able to endure conventional hemodialysis because his blood pressure was low. Thus, he received continuous renal replacement therapy in the intensive care unit, which however could not be performed effectively secondary to frequent hypotensive episodes despite the use of inotropic therapy. Thus, we selected a more conservative therapeutic approach using bilateral indwelling pleural catheters. However, the volume of drainage was >600 mL/d over 2 weeks. Thus, we decided to empirically administer a course of prednisolone (1 mg/kg/d, initial dose of 50 mg/d) to manage pleural effusion in this patient who was refractory to conventional treatment, given the possibility of nonspecific pleuritis.

**Table 1:** Findings of pleural fluid analysis

	Right	Left
Total protein (g/dL)	2.6	2.4
Albumin (g/dL)	1.5	1.7
Glucose (mg/dL)	186	150
Lactate dehydrogenase (IU/L)	166	160
Adenosine deaminase (IU/L)	11	19
Red blood cells (/mm^3^)	150	200
White blood cells (/mm^3^)	50	180
Neutrophils (%)	4	2
Lymphocytes (%)	82	78
Mononuclear cells (%)	13	15
Eosinophil (%)	1	1
Bacterial culture	(‐)	(‐)
Acid‐fast bacilli	(‐)	(‐)
Tuberculosis polymerase chain reaction	(‐)	(‐)
Cytology	(‐)	(‐)
Serum total protein (g/dL)	4.3	
Serum albumin (g/dL)	2.2	
Serum glucose (mg/dL)	202	
Serum lactate dehydrogenase (IU/L)	553	

## 4 OUTCOME AND FOLLOW‐UP

**Figure 3:**
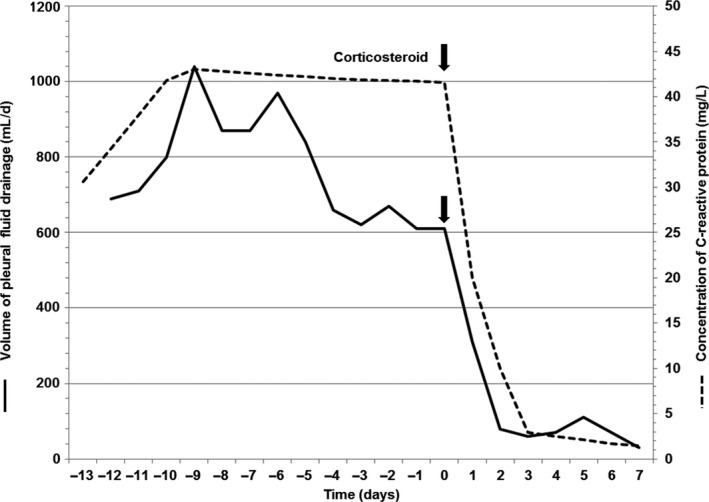
Change of volume of pleural fluid drainage and C‐reactive protein concentration before and after treatment with corticosteroid

The volume of drainage rapidly decreased although albumin did not be administered, and the serum CRP level also returned to normal a week after the administration of prednisolone (Figure 3). We removed the indwelling pleural catheters and tapered the administration of prednisolone over 4 weeks. On the 39th hospital day, he was discharged without any symptoms, and the serum creatinine and CRP levels had decreased to 2.23 mg/dL (estimated glomerular filtration rate; 28 mL/min/1.73 m^2^) and 1.50 mg/L, respectively. At his 4‐month follow‐up, no recurrence of pleural effusion was observed, and his renal function showed no significant change (serum creatinine; 2.43 mg/dL, estimated glomerular filtration rate; 25 mL/min/1.73 m^2^), and he did not require the initiation of dialysis.

## 5 DISCUSSION

Uremic pleuritis results from chronic fibrinous inflammation caused by unknown pathogenesis. The typical patient with uremic pleural effusion is one who has been undergoing hemodialysis or peritoneal dialysis over 1‐2 years.^2^, ^6^, ^9^, ^10^ Uremic pleural effusion per se has been reported in approximately 24% of patients with terminal uremia who require renal replacement therapy or on dialysis.^1^, ^2^, ^3^, ^6^, ^9^ However, the incidence of pleural effusion in patients with CKD not receiving dialysis is significantly lower than that in patients with more severe disease necessitating renal replacement therapy.^2^, ^3^ Moreover, although uremic pleural effusion is not related to the severity of uremia and may occur at any time in the course of kidney dysfunction (from shortly after uremia to several years later),^6^ it has rarely been reported in patients with CKD who do not receive dialysis, even among those in whom the effusion is expected to resolve after the institution of hemodialysis.

In our patient, the pleural effusion appeared before the initiation of maintenance hemodialysis and was not attributable to other etiologies including congestive heart failure, infections such as tuberculosis, malignancies, or connective tissue diseases. Additionally, the pleural fluid was an exudate with a paucity of nucleated cells that were predominantly lymphocytes, and his serum CRP levels were increasing without evidence of infection. Therefore, we speculated that an inflammatory process such as uremic pleuritis could cause exudative pleural effusion. However, the patient refused a pleural biopsy, and even continuous renal replacement therapy could not be effectively instituted secondary to the occurrence of frequent hypotensive episodes. We thought the difficulty of treatment of this patient was due to massive pleural effusion and subsequent deficient intravascular volume. Furthermore, the volume of fluid drained via the indwelling pleural catheters did not decrease over 2 weeks. Therefore, we thought that reducing the production of pleural fluid may be beneficial to manage the patient, and finally decided to administer corticosteroid empirically to control nonspecific inflammation. This strategy produced an immediate response with reduction of the volume of the effusion and improvement in dyspnea although we could not confirm his case as uremic pleuritis due to lack of pleural biopsy.

The management of patients with uremic pleural effusions involves various options including intensified continued hemodialysis, repeated thoracentesis, chest tube drainage, pleurodesis, and surgical pleurectomy.^6^, ^7^, ^9^, ^11^ However, patients may be unresponsive to these therapies,^2^, ^6^, ^7^ and to date, the optimal treatment in refractory cases has not been established. Only a few reports in the literature have described the successful use of corticosteroid therapy to refractory uremic pleural effusion.^12^, ^13^ However, the patients reported in previous studies had been on hemodialysis for a range of number of years, for example 5‐13 years before the development of pleural effusion. In our case, the patient had not been receiving dialysis before admission, and renal replacement therapy was not required after the resolution of the pleural effusion. To our knowledge, this is the first report to describe the efficacy of corticosteroid to treat exudative pleural effusion in a CKD patient not receiving dialysis. It must be mentioned, however, that pleural biopsy could not be performed, and whether the effusion was uremic in origin remains unknown.

In conclusion, we need to consider uremic pleural effusion and pleuritis in differential diagnosis of exudative lymphocyte predominant pleural effusion in patients with CKD not receiving dialysis. In addition, corticosteroid therapy may be a therapeutic option to treat refractory exudative pleural effusion in these patients, in whom conventional treatments are not applicable after ruling out infections and malignancies with pleural biopsy.

## CONFLICT OF INTEREST

None declared.

## AUTHOR CONTRIBUTIONS

HK: conceived the idea. HMS: wrote the manuscript with support from Hyunwoo Kim. MK: collected clinical data. The final manuscript was evaluated and approved by all authors.
